# Analytical quality by design approach to develop an eco-friendly RP-HPLC method for estimation of irbesartan in chitosan polymeric nanoparticles: forced degradation studies and assessment of *in vitro* release mathematical modelling

**DOI:** 10.1039/d4ra03952a

**Published:** 2024-07-12

**Authors:** Sinchana B Gopalaiah, Kavitha Jayaseelan

**Affiliations:** a Department of Pharmaceutical Analysis, SRM College of Pharmacy, SRM Institute of Science and Technology Kattankulathur, Chengalpattu District – 603203 Tamil Nadu India kavithaj@srmist.edu.in (+91) 9094903309

## Abstract

Irbesartan is an angiotensin converting enzyme blocker, primarily utilized for the management of hypertension and the mitigation of diabetic nephropathy progression. The present study introduces rapid, robust and environmentally sustainable reverse phase high performance liquid chromatography (RP-HPLC) validated under the analytical quality by design (AQbD) framework according to ICH guidelines. Utilizing a central composite design, the method's systemic optimization was achieved, ensuring reproducibility and accuracy. Chromatographic separation was accomplished utilizing an ethanol and sodium acetate buffer (60 : 40 v/v) isocratic mobile phase system on a zorbax sb C_18_ column, with a flow rate of at 0.6 mL min^−1^. Studies on forced degradation outlined stability of irbesartan and its degradation processes, enhancing our understanding of its chemical robustness under varied conditions. Complementing the green chemistry paradigm, the method's environmental impact was critically assessed, affirming its alignment with sustainability objectives. The validated method proved pivotal in determining the percent entrapment and loading efficiency of the formulated nanoparticles and holds potential for application in biological matrices. Furthermore, the encapsulation of IRB within chitosan nanoparticles was explored to assess release kinetics and enhance bioavailability. This study not only advances the analytical sciences by merging eco-friendly practices with method development but also broadens the applicative landscape of HPLC methodologies in drug delivery research.

## Abstract 2

Stability indicating eco-friendly AQbD assisted RP-HPLC method for the estimation of Irbesartan chitosan nanoparticles and assessment of *in vitro* release mathematical modelling.

## Graphical abstract

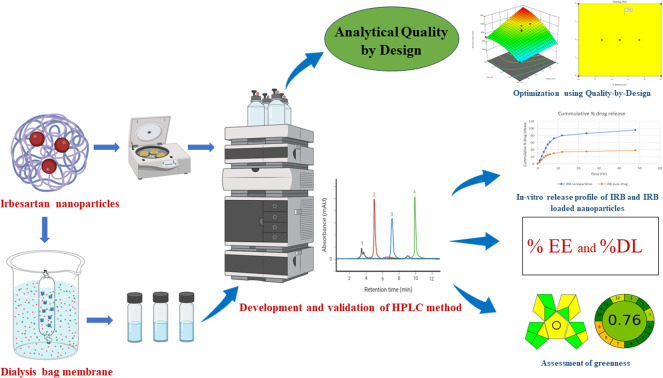

## 1. Introduction

**Fig. 1:**
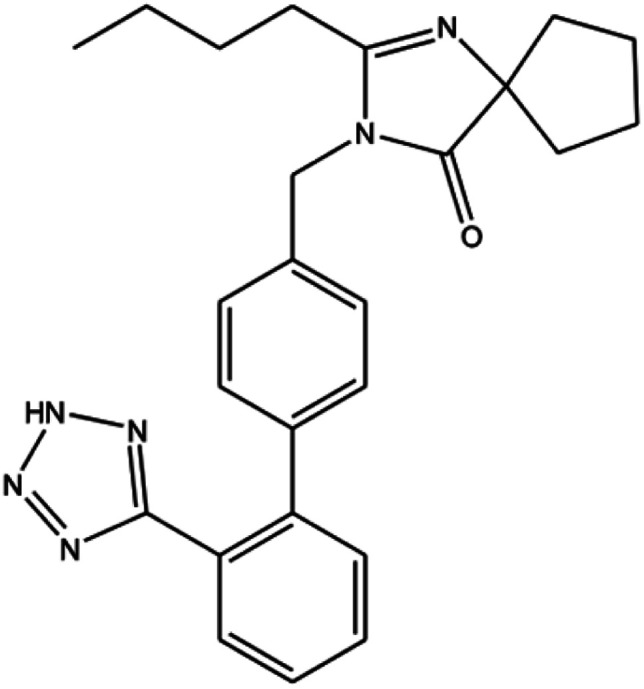
Structure of irbesartan.

Irbesartan (IRB) is primarily employed to treat cardiac insufficiency, cardiac arrhythmia, and hypertension and has been classified as a class II drug under the biopharmaceutic classification system (high permeability and low solubility) (Fig. 1).^1,2^ The therapeutic effects of IRB are overshadowed by its poor aqueous solubility.^3^ IRB chitosan nanoparticles were created with great attention to enhance their bioavailability and dissolution rate.^4,5^ IRB's low solubility is due to first-pass metabolism, in order to get around these issues we have created a nanocarrier technology that lowers first-pass metabolism and boosts solubility. Nonetheless, a number of nanocarrier systems, including nanostructured lipid carriers, solid lipid nanoparticles were created to encapsulate the IRB.^6–10^ However, screening a wide range of components is necessary to formulate lipid-based formulations. In addition to this process, these components are linked to quality problems like polymorphic changes in lipids, various colloidal species, gelation, the presence of supercooled melts and sterilisation stability.^11,12^ Furthermore, biodegradable polymer, such as chitosan, was used in the formulation of the polymeric nanoparticles to circumvent these problems.

One of the most prevalent polysaccharides in nature is chitosan (CS). Chitin can be partially deacetylated in an alkaline solution to get it.^13^ Because of its inherent qualities, which include biodegradability, biocompatibility, nontoxicity, non-immunogenicity, non-carcinogenicity, and antibacterial qualities, CS is garnering increased interest for use as a drug delivery vehicle.^14^ Furthermore, CS is positively charged and has properties that enhance absorption.^15^ The most significant characteristic of CS is its solubility in the majority of organic acidic solutions with a pH of less than 6.5 and its ease of crosslinking with agents like glutaraldehyde, tri-polyphosphate and sodium salt of poly-aspartic acid to create nanoparticles. CS is a perfect drug delivery vehicle for the hydrophobic drug like IRB.^16^

The reported high performance liquid chromatography (HPLC) techniques for assessing IRB identifies several drawbacks, such as the employment of non-biodegradable solvents^17,18^ and prolonged analysis durations.^19^ Additionally, Table 1 highlights other methods where the employed mobile phases are not environmentally sustainable. Rishab *k* Dagaria and colleagues developed a HPLC methodology using a complex solvent system composed of potassium dihydrogen phosphate (pH 3.5): acetonitrile: tri-ethylamine in the ratio 80 : 20 : 0.1 v/v.^21^ Similarly, R. A. Mhaske *et al.* devised a RP-HPLC method to quantify the levels of irbesartan, losartan, hydrochlorothiazide, and chlorthalidone simultaneously using a gradient technique that begins with the mobile phase containing 0.05 M sodium dihydrogen phosphate buffer and acetonitrile in a 70 : 30 v/v ratio.^22^ Furthermore, Reem Youssef and colleagues developed an HPLC method utilizing acetonitrile : phosphate buffer at pH 3 (40 : 60 v/v) as mobile phase to determine valsartan, losartan, and irbesartan, with irbesartan eluting at 15.82 minute.^20^ These aforementioned methodologies employ non-biodegradable organic phases, contributing to their time-intensiveness and environmental detriment. A cost-effective UV-visible spectroscopic methods are developed for the quantification of irbesartan in pharmaceutical dosage forms, bulkand in biological matrix, but the spectroscopic methods developed are not robust and lacks sensitivity.^25–31^ Additionally none of the methods have used eco-friendly solvents to develop the method.

**Table 1:** Comparison of the HPLC methods published with the suggested method

Sl. no	Mobile phase used	Retention time (min)	Limitations of the reported method	Benefits of the proposed method	Ref.
1	Acetonitrile : sodium acetate buffer ph adjusted to 3.5 using ortho phosphoric acid (55 : 45 v/v)	4.8	• Increased amount of organic phase is used	• The AQbD technique, which was not employed by any of the published approaches, was used to build the proposed method	17
			• Forced degradation studies are not carried out		
			• High injection volume	• Proposed method is environmentally friendly	
2	Potassium dihydrogen phosphate (pH 3.0) : acetonitrile, (70 : 30 v/v)	9.6	• Degradation studies were studied using MS detector which increased the capital and operating cost	• Studies on forced degradation using different stressing agents have been conducted, demonstrating the method's specificity	19
3	Potassium di-hydrogen orthophosphate : acetonitrile, (55 : 45 v/v)	5.2	• High injection volume	• Cost-effective	18
			• Increased amount of organic phase is used	• Simple method	
				• Complete validation of the optimized method	
4	Acetonitrile : phosphate buffer pH 3 (40 : 60 v/v)	15.82	• Long analysis time	• Accurate and robust method	20
			• Volume of organic phase used is more		
			• Forced degradation studies are not carried out		
6	Potassium dihydrogen phosphate buffer 0.05 M (pH 3.5) : cetonitrile : TEA (80 : 20 : 0.1 v/v)	3.8	• Forced degradation studies are not carried out		21
			• Mobile phase employed is complicated		
7	0.05 M sodium dihydrogen phosphate buffer : acetonitrile (gradient mode)	13.22	• Increased volume of organic phase is used		22
			• Long analysis time		
8	Sodium acetate buffer pH 4.0 : acetonitrile (30 : 70 v/v)	6.2	• Injection volume is more		23
			• Long analysis time		
			• Increased amount of organic phase is used		
10	Ammonium acetate buffer (10 mM; pH 4.0) : acetonitrile (40 : 60 v/v)	1.2	• High injection volume		24
			• High volume of organic phase is used		

One factor at a time (OFAT), a conventional technique development strategy, has been used in the reported methods. It takes longer to complete OFAT and is more challenging to comprehend the important characteristics. Inadvertent mistakes in an experimental study can result in time loss, substantial financial costs, and decreased empirical accuracy.^32^ Analytical quality by design (AQbD), is based on design experiment concepts and quality risk management. It facilitates a thorough understanding of all potential risks and the concurrent interactions among the method's variables. It primarily aims on the risk-based important characteristics those influence the method's performance in order to generate a novel, reliable analytical technique.^33^

In pharmaceutical analysis, HPLC stands as the primary analytical technology, performing an essential function in bulk and dosage form quality control processes and it proves to be reliable approach in terms of precision, robustness and sensitivity.^34^ To establish a ‘design space’ for developing robust analytical procedures quality by design (QbD) techniques leverage design of experiment (DoE). Within this design space, changes in technique parameters have minimal impact on outcomes, ensuring intrinsic stability during the development phase. The food and drug administration (FDA), which emphasises the methodical integration of quality into both in product and process development through risk assessment, has actively promoted QbD concepts. This process is not the same as the conventional testing-based retrospective quality assessment. Increasingly, QbD and its concepts are being used in the creation of analytical procedures in an effort to improve method efficacy and stability.^35^ Central composite design (CCD) is a widely employed methodology for optimizing HPLC techniques.^36^

The adoption of sustainable HPLC techniques has become a priority in drug testing, aiming to safeguard operator health while also preserving the environment. A lot of solvents are used and an enormous amount of waste is made when chromatographic instruments are used to check the quality of pharmaceuticals.^37^ Despite their advantages, acetonitrile and methanol pose significant environmental risks. Acetonitrile is highly flammable and quickly vaporises, posing toxicity concerns.^38^ The scientific community has become more interested in Green Analytical Chemistry (GAC).^39^ By eliminating or reducing dangerous compounds from analytical processes, GAC aims to enhance environmental and health compatibility without sacrificing analytical performance.^40^ The incorporation of Green Analytical Chemistry principles into AQbD framework ensures the efficacy of a chromatographic method by conducting thorough risk assessments and adhering to environmentally sustainable criteria.

Table 1 contrasts existing methods of estimating IRB with the proposed approach, underscoring the necessity for a rapid, eco-friendly, highly sensitive, QbD supported, and cost-effective method to measure extensive quantities of IRB and its nano-formulations. Despite the diversity in techniques for assessing IRB, they often suffer from the use of toxic, non-biodegradable mobile phases. Moreover, the majority of documented HPLC techniques are developed to estimate IRB in combination with other pharmaceutical compounds, and currently, no robust, eco-friendly QbD-supported HPLC methodology exists for quantifying IRB as a standalone drug molecule. This study introduces an innovative chromatographic approach that effectively combines AQbD and GAC principles by employing ethanol as the solvent. Ethanol is considered a less hazardous solvent compared to acetonitrile and methanol, aligning with environmentally sustainable practices. The approach is both economical and environmentally benign for developing novel products, notably nano-formulations, which are becoming popular in the pharmaceutical industry. The discovered critical method parameters (CMPs) were optimised using the CCD. The optimised approach was validated using the ICH guidelines. This technique was also used to determine the pace at which IRB-loaded polymeric nanoparticles are released.

## 2. Materials and methods

### 2.1 Chemicals and equipments used

The irbesartan (98%) was purchased from Yarrow Chem Products (Mumbai, India). Ethanol was purchased from Hayman Group Ltd, (Witham, UK). Sodium hydroxide (NaOH) and analytical grade sodium acetate anhydrous were purchased from SRL (Maharashtra, India). 30% hydrogen peroxide (H_2_O_2_), hydrochloric acid (HCL) and glacial acetic acid were procured from Rankem, New Delhi, India. The ELGA Lab Water system was used internally to obtain HPLC grade water. Agilent HPLC 1220 Infinity II system (California, USA) was used to develop the method which is built with a pulse-free solvent delivery system consists of two-channel, dual-plunger in-series pumps. Thermal sensored column oven, intelligent optical sensored autosampler and Diode Array Detector.

### 2.2 Chromatographic conditions and software used

Zorbax sb column (C_18_, 150 mm × 4.6 mm i.d, 5 μm) was used to develop the RP-HPLC method for estimating IRB using HPLC grade ethanol and sodium acetate buffer (pH 3.5) as mobile phase. The wavelength used was 240 nm, and the sample injection volume was 10 μL. The experimental design was developed using the trial version 12 of Design-Expert® software from Stat-Ease Inc., which has its headquarters in Minneapolis, USA., which emphasised on optimising several critical parameters and establishing the experimental design space. HPLC Agilent Chem Station (Version B) software was utilised for both data gathering and processing.

### 2.3 Preparation

#### 2.3.1 Preparation of mobile phase

Sodium acetate buffer was prepared by dissolving 13.6 g of anhydrous sodium acetate in 500 mL of HPLC grade water and glacial acetic acid was used to adjust the pH to 3.5 and then the volume was made up to 1000 mL using HPLC grade water.

#### 2.3.2 Stock and working standard solution preparation

Stock and working solutions of the drug IRB were prepared by dissolving the drug in the solvent ethanol. Primary stock solution, 1 mg mL^−1^ (1000 μg mL^−1^) concentration was obtained by dissolving 10 mg of IRB in 10 mL of ethanol. Subsequently, to produce a working solution of IRB, 1 mL of the primary IRB stock solution was diluted with 10 mL of ethanol. The working IRB solution was then serially diluted with ethanol to obtain the final concentrations of 80, 90, 100, 110, and 120 μg mL^−1^.

### 2.4 Chromatographic method development assisted by quality by design

#### 2.4.1 Risk assessment

Research works performed on risk assessment were used to analyse how different components affected the quality target method profile (QTMP) (Table 2). It is employed to categorise potential problem sources in order to identify and manage the variables resulting in failures, errors, defects or variances.^41^ In the course of the analysis, significant data is extracted from risk assessment studies based on the likelihood and risk associated with the various components by allocating low, medium, and high risk scores to each element. Six criteria were selected for screening and prioritisation, and they were divided based on their risk scores. Two of them were selected as CMPs in order to use experimental designs to optimise critical analytical attributes (CAAs) in a systematic manner.^42^ Preliminary screening and prior experience established the ethanol volume and flow rate as CMPs. The intention of the ethanol ratio was to enhance peak area and the number of theoretical plates in order to maximise column efficiency. The mobile phase's flow rate across the HPLC column affects the sensitivity, resolution, and length of the study. In order to balance these effects, flow rate optimisation is crucial. Peak area (R1), tailing factor (R2), retention time (R3) and theoretical plates (R4) were identified as CAAs influencing QTMP in order to achieve an effective method.^35,43,44^ The strategies implemented aimed at enhancing the theoretical plate count to optimize column efficiency. To achieve favourable peak shapes, the goal of minimising peak tailing was pursued. Table 3 displays the CMPS and their scores in relation to CAAs.

**Table 2:** Hypothesized quality QTMP for the HPLC analysis of irbesartan

Method parameters	Target	Justification
Analyte of interest	Irbesartan	Development of an HPLC method for the detection of IRB, an active analyte in samples
Sample	IRB loaded chitosan nanoparticles	It is critical to develop an HPLC method for detecting IRB in polymeric nano-formulations
Method type	RP-HPLC	In RP-HPLC technique, a lipophilic stationary phase helps hold on to most of the drug molecules that are very lipophilic. IRB has a substantial degree of lipophilicity, with a log *p* value of 4.1. Hence the RP-HPLC will be more effective
Instrument requirement	Binary pump with PDA detector	The binary pump offers exceptional precision in blending mobile-phase liquids, while the PDA detector identifies degradation products at various wavelengths
Sample characteristic	Liquid sample	In order to quantify and detect the analyte using reverse phase chromatography, it is necessary to prepare it in liquid form so that it can mix well with the mobile phase
Standard preparation	Standard dilutions of drug	The standard dilutions were generated using a mobile-phase mixture for drug separation
Sample preparation	Weighing, dissolving with solvents	To obtain a stock, it is necessary to accurately weigh an appropriate quantity of drug and mix it with the solvents for preparing samples. Subsequently, it is necessary to do serial dilutions following the sonication of the primary stock solution to ensure the complete dissolution of drug
Application of the method	For the determination of %EE and %DL	The approach should have the ability to analyse IRB in large quantities and in chitosan nanoparticles

**Table 3:** Risk assessment parameters for developing the robust HPLC method^a^

HPLC method parameters of IRB							
CAAs	%Of ethanol	Column temperature (25–40 °C)	Flow rate	pH of acetate buffer	Type of flow	Volume of injection	Column dimen-sion
Peak area	+	—	+	+	—	—	0
Retention time	+	0	+	+	0	—	0
Tailing factor	+	—	0	—	—	—	0
Theoretical plate number	+	—	+	+	0	—	0

a High risk (+), middle risk (0), low risk (−).

#### 2.4.2 Optimization of HPLC method using CCD

The response surface method was combined with a CCD to further enhance the suggested HPLC procedure. In this design, the centre points are supplemented with a series of axial points called star points. This design makes it easy to approximate first- and second-order terms. Following the completion of the risk analysis, preliminary trials were carried out to identify the suitable ranges of selected method attributed. A DoE strategy, CCD was applied to know the effect of independent parameters on dependent responses. This study was designed with design expert software version 12. The design includes axial points and centre points, which aid in continuous experimentation and the estimation of quadratic or higher order models. In experimental designs, CCD improves validity and reproducibility. Table 4 contains the experimental variables. The optimisation of the procedure involved the use of dependent variables like retention time, theoretical plate numbers, tailing factor, peak area and independent factors like flow rate and ratio of mobile phase. The tailing factor indicates the method's efficiency, peak area signifies the concentration of the drug, while the retention time of a medication quantifies its capacity to separate. The performance of the procedure and the suitability of the mobile phase are indicated by theoretical plates. The results of all examined situations were fitted to quadratic equations using the multiple regression technique in order to choose the best fit model. Moreover, analysis of variance (ANOVA) was used to study each factor's relative importance to the responses. Additionally derived polynomial equations were helpful in producing perturbation, contour, and 3-dimensional response surface plots.

**Table 4:** Measured responses to a two-factor, thirteen-run central composite experimental design

Runs	Factors		Responses			
	Ethanol (%v/v)	Flow rate (mL min^−1^)	Peak area of IRB	Retention time of IRB	Tailing factor of IRB	Theoretical plate numbers of IRB
1	60	0.3	1509	4.1	1.31	5012
2	55	0.8	1214	2	1.14	5822
3	60 #	0.6	1347	2.8	1.21	6022
4	62	0.6	1488	2.7	1.23	6449
5	59	0.6	1444	2.5	1.28	5612
6	60	0.8	1323	1.7	1.10	6232
7	60 #	0.6	1427	2.8	1.27	6512
8	60 #	0.6	1644	2.8	1.22	6214
9	65	0.4	1620	4	1.32	5482
10	60 #	0.6	1425	2.9	1.24	6018
11	60 #	0.6	1522	2.7	1.31	6272
12	65	0.8	1325	2.1	1.12	6832
13	55	0.4	1617	3.8	1.29	5432
Factors	Levels					
	Low (−1)		Middle (0)		High (+1)	
Mobile phase ratio (v/v)	55 : 45		60 : 40		65 : 35	
Flow rate mL min^−1^	0.4		0.6		0.8	

### 2.5 Validation of the method

The long-term effectiveness and reliability of an analytical techniques in field like clinical, toxicological investigations, research, product development, quality assurance, process control as well as regulatory goals, depend on their validation. This is important because consistent target variability enhances the repeatability and dependability of data quality by enabling the identification of unusual behaviour in analytical data in day to day applications like quantitative analysis. It also demonstrates the stability, predictability, and repeatability of an analytical method. Thus, in accordance with ICH guidelines, the HPLC technique developed was validated for system suitability, linearity, precision, accuracy, limit of quantification (LOQ) and limit of detection (LOD).

#### 2.5.1 Quality control samples preparation

Quality control samples were generated at three distinct concentration levels: lower quality control (LQC, 80 μg mL^−1^), middle quality control (MQC, 100 μg mL^−1^) and high quality control (HQC, 120 μg mL^−1^). The samples refrigerated until further analysis. Prior to chromatography, samples prepared were filtered through a syringe filter of pore size of 0.22 μm.

#### 2.5.2 System suitability

**Fig. 2:**
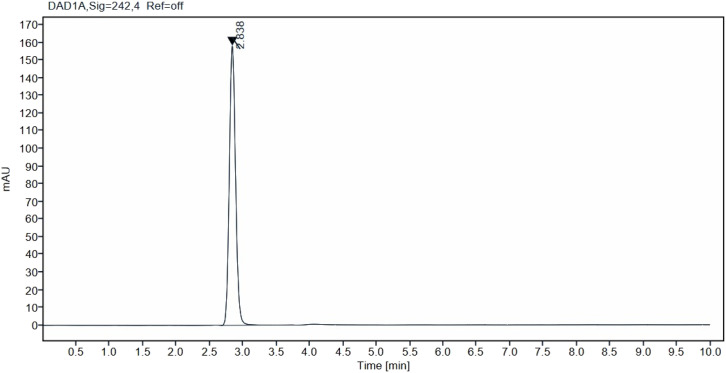
Standard chromatogram of irbesartan (50 μg mL^−1^) at Rt 2.83 min.

Column efficiency, and repeatability of the chromatographic system was checked ensure the system suitability to make sure it is suitable for the analysis. 6 injections of 50 μg mL^−1^ solutions were injected to the instrument to carry out the test. The percent relative standard deviation (%RSD) of the tailing factor, retention time, theoretical plates and peak area were calculated and compared to the regulatory limits.^45,46^Fig. 2 illustrates the standard IRB chromatogram.

#### 2.5.3 Linearity

The samples prepared at concentrations between 80 and 120 μg mL^−1^ from the working standard solution were used to assess the linearity for the method developed. The linearity graph was plotted taking concentration of IRB (μg mL^−1^) against peak area (mAU) to determine regression coefficient (*R*^2^), slope, and regression equation.^47^

#### 2.5.4 Limit of detection and limit of quantification

LOQ and LOD were determined in order to carefully evaluate the sensitivity of the method. The LOQ was found using the formula LOQ = 10 *σ*/*S*, and the LOD was found using equation LOD = 3.3 *σ*/*S*. *S* is the calibration curve's slope in both cases, and *σ* denotes each response's standard deviation.^48^

#### 2.5.5 Accuracy

The method's accuracy was assessed by evaluating the absolute recovery of IRB across its low, medium, and high quality control concentrations. This experiment was conducted in triplicate, and for each sample, mean values, to ensure accuracy within predetermined thresholds, %RSD and recovery were calculated.^49^ The recovery percentage was calculated using the eqn (1) which is given below.1
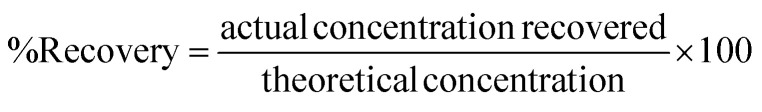


#### 2.5.6 Precision

The precision of the HPLC method under scrutiny elucidates the uniformity of outcomes across numerous separate aliquots of identical concentration. The suggested method's intraday (repeatability) and interday (reproducibility) were examined by injecting 3 prepared quality control samples of IRB on the first day and second day under similar conditions for experimentation. %RSD values were calculated.^48^

### 2.6 Forced degradation studies

The capacity of method to forecast stability was examined by treating standard solutions (1000 μg mL^−1^) to stress conditions, such as alkali, acid, heat and oxidation. Deterioration was indicated by the appearance of new peaks or a decrease in peak area, and the recovery percentage was used to calculate the percentage of degradation. Sample solutions were treated with sodium hydroxide (0.01 M) and hydrochloric acid (0.01 M), simulating basic and acidic hydrolysis. These solutions were then allowed to stand at room temperature for six hours, and then they were neutralised. Sample solutions for oxidation degradation were prepared treating with 0.3% hydrogen peroxide solution and left at room temperature for six hours while being protected from light. The reference solution was kept in hot air oven at 80 °C for six hours in order to quantify thermal degradation. The final solutions were then injected into HPLC under ideal chromatographic conditions after being diluted to 50 μg mL^−1^ with ethanol.^50^

### 2.7 Method applicability

#### 2.7.1 Formulation and characterization irbesartan loaded chitosan nanoparticles

The synthesis of chitosan nanoparticles is a reaction based on an ionic reaction between a negatively charged tripolyphosphate (TPP) solution and a positively charged chitosan solution. Concentration of 2 mg mL^−1^ of TPP was first dissolved in distilled water and CS was dissolved at a concentration of 3 mg mL^−1^ in a solution of 1% aqueous acetic acid. The IRB was directly dissolved in TPP solution prior to the CS nanoparticle production. The prepared TPP solution contain IRB was mixed with CS solution while being stirred by magnetic stirrer at 1000 rpm at room temperature, the obtained suspension was stirred for 30 minute to promote further nanoparticle crosslinking.^16^ The Malvern Nano ZS (Malvern Instrument Ltd., UK) was used to determine the particle size and polydispersity index (PDI) of the nanoparticles. Additionally, the ultracentrifugation technique was utilized to quantify the amount of IRB encapsulated within the nanoparticles. To sum up, the nanoparticles were centrifuged at 20 000 rotation per minute for 15 minute, the pellet that was produced was then collected and subjected to an advanced HPLC technique for analysis.^50^ The following eqn (2) and (3) were used to compute the entrapment efficiency (%EE) and drug loading (%DL).2
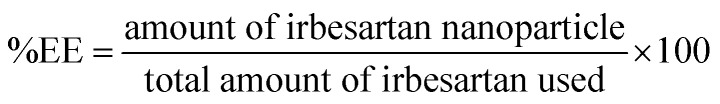
3
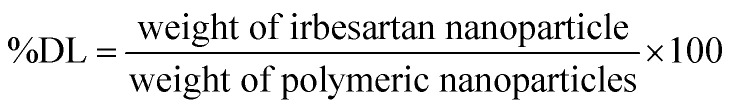


#### 2.7.2 *In vitro* release profile

IRB-loaded polymeric nanoparticles' *in vitro* release pattern was investigated by dialysis bag method. A dialysis membrane with a pore size of 2.4 nm and a molecular weight cut-off between 12 000 and 14 000 kDa was used in the investigation (Hi-Media, Mumbai, India). Before the release experiments, the membrane was immersed in distilled water for the whole night. The release medium for the release tests was a 7.4 pH phosphate buffer containing 40% ethanol. Organic co-solvents, such as ethanol, are frequently added to aqueous dissolving medium in accordance with USP criteria in order to improve solubility and guarantee sink conditions. The experimental unit consisted of the donor and recipient chambers. A temperature of 37 ± 0.5 °C was maintained continuously for the diffusion cell. After every 0.5, 1, 2, 3, 4, 6, 8, 10, 12, 24, and 48 hours, a one millilitre sample was removed from the receiver chamber and replaced with an equal volume of brand-new receiving medium. Using HPLC analysis, the IRB's cumulative release profile over time was ascertained.^6^

## 3. Results and discussions

### 3.1 HPLC method development and optimization using AQbD

The QTMP was to develop the method with the goal of measuring the IRB within a specific design space while applying eco-friendly analytical concepts. Furthermore, this technology must be economical, user-friendly, and reproducible, making it suitable for routine usage in quality control laboratories.

### 3.2 Mobile phase selection and preliminary HPLC method development studies

Identifying an appropriate mobile phase presented major challenges in analytical chemistry, especially when following Green Analytical Chemistry principles. The organic phase in this investigation was selected based on the GSK solvent selection criteria. Biodegradable ethanol emerged as the best option for toxic solvents like methanol and acetonitrile. Using the *G* values from the green solvent selection tool (GSST), which can be found online at https://green-solvent-tool.herokuapp.com/, the new HPLC method effectively swapped out acetonitrile and methanol for ethanol.

*G*_ethanol_ = 6.6, category scores: waste = 4.2, health = 8.9, environment = 6.7, safety = 7.7

*G*_acetonitrile_ = 5.8, category scores: waste = 2.8, health = 5.9, environment = 8.9, safety = 7.7

*G*_methanol_ = 5.8, category scores: waste = 4.0, health = 4.9, environment = 8.4, safety = 7.1

**Fig. 3:**
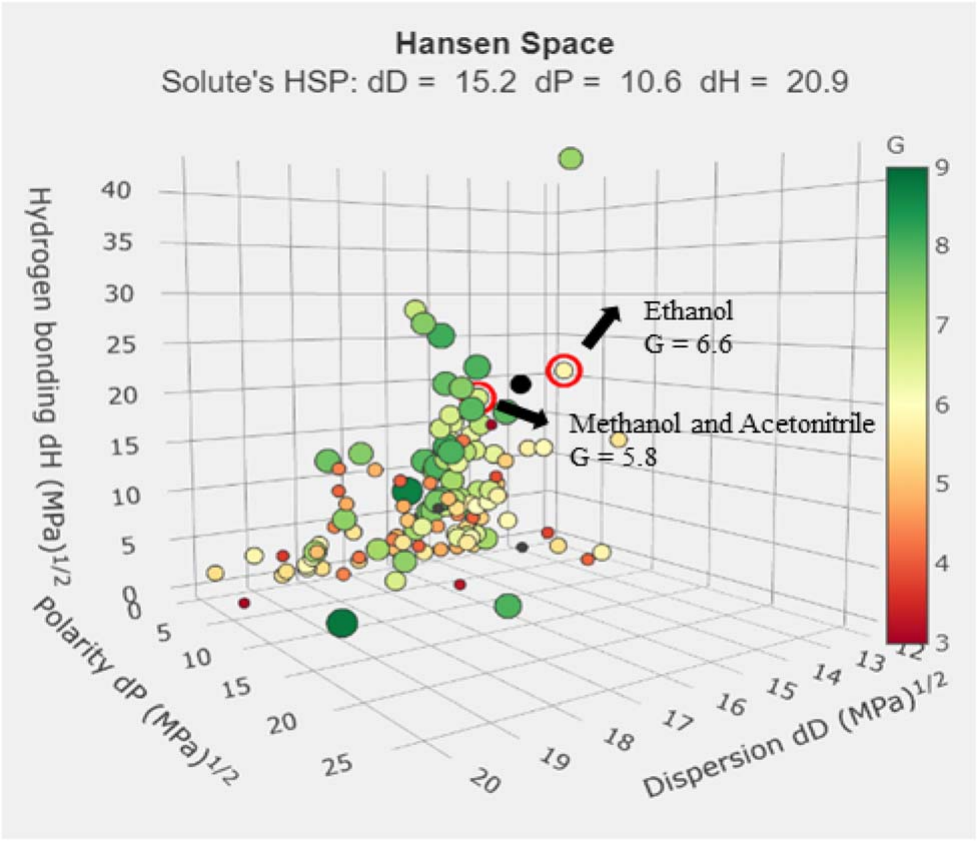
Hansen space for the selection of solvents for the analysis.

Compared to conventional HPLC procedures, the novel method worked to find out sustainable solvents which are having high *G* value. The GSST tool, available for free online, makes it easier to evaluate solvents using the solvent sustainability guidelines by GSK. Fig. 3 depicts the Hansen space for solvent selection. During the initial RP-HPLC development with water and ethanol as the mobile phase, we were unable to achieve the required peak shape. We evaluated several buffers, including formate, acetate, citrate and phosphate. Notably, the combination of phosphate buffer and ethanol did not result in acceptable peaks. However, we made a breakthrough with a combination of ethanol and acetate buffer, which produced better results. The key problem in designing the approach was retaining IRB in the column. Adjusting the ratio to 60 : 40 (ethanol : acetate buffer, v/v) resulted in fast elution of IRB at 2.8 minute, which aligns with GAC principles by decreasing waste. We also tested several flow rates (0.4, 0.6, and 0.8 mL min^−1^) to achieve shorter retention times, less peak tailing, higher theoretical plate numbers, and better technique efficacy. The IRB's retention time increased as the flow rate increased from 0.4 to 0.8 mL min^−1^. The best chromatograms were obtained with a mobile phase of 60 : 40 (v/v) ethanol and acetate buffer. Subsequently, we employed the AQbD principles in the method to ascertain the method operable design region (MODR), wherein the chromatographic conditions were fine-tuned primarily by the ethanol volume and flow rate.

#### 3.2.1 Risk assessment studies

To ensure compliance with strict quality standards, detailed risk assessment studies are required to determine the many causes and sub-factors contributing to variability, as well as their potential impact on important analytical properties. Table 3 summarizes the results of a study of key components used to determine risk. Drawing on a thorough examination of relevant literature, the study assessed the risk levels associated with each procedural parameter, taking into account a variety of input elements and determining their importance and likelihood. The risk assessment matrix clearly summarised these evaluations. Notably, the mobile phase ratio and flow rate emerged as major issues. Whereas the flow rate exert medium risk on tailing factor but it exerts high risk on the other three CAAs selected. While the assessment of column dimensions indicated a moderate level of danger. On the other hand, we assessed characteristics like column temperature, injection volume, and flow type as low-risk with minimal anticipated impact on method performance. We developed a systematic framework for continuous improvement, leveraging the best results from screening and risk assessment efforts. This included improving processes using experimental designs, modelling, and response surface approaches to produce the best results within the analytical design space.

#### 3.2.2 Optimization of the developed method using CCD

We employed CCD in our study to optimise the chromatographic conditions for the separation of IRB in large-scale quantities. The variables under scrutiny encompassed the proportion of the mobile phase and the rate of flow. We conducted thirteen experimental trials, incorporating five central points within the design framework (Table 4). The evaluation focused on crucial attributes such as retention time, theoretical plate numbers, peak area and tailing factor. The CCD found the best chromatographic conditions, with a score of 1000 for desirability, which meant that the mobile phase ratio should be 60 : 40% v/v and the rate of flow should be 0.6 mL min^−1^.

#### 3.2.3 Statistical evaluation of the method using DoE software

**Fig. 4:**
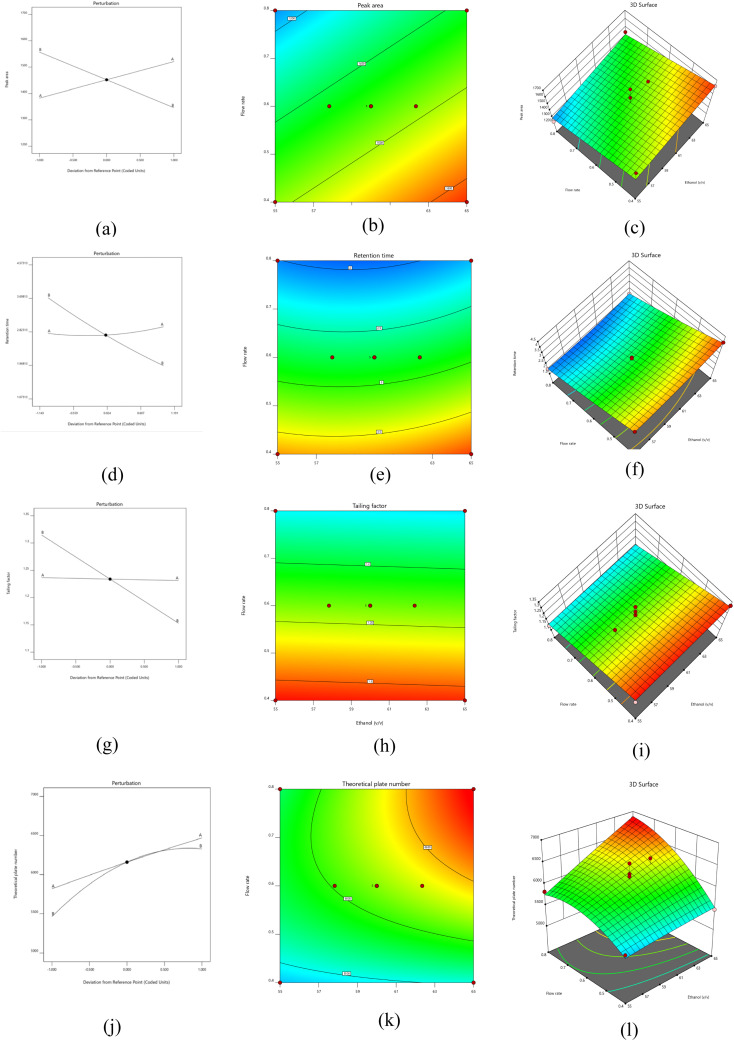
Perturbation plot (a, d, g, and j), contour plot (b, e, h, and k), and 3-D response plot (c, f, I, and l) for peak area, retention time, tailing factor and theoretical plate number of irbesartan respectively.

After limiting the variables and performing graphical and numerical optimisation, it was imperative to perform a sequential statistical analysis of the experiment's results. We conducted ANOVA analysis of experimental data from multiple runs using diverse kinetic models such as quadratic, cubic, second-order linear models. Notably, the linear and quadratic models showed strong associations with peak and area, tailing factor, retention time, and theoretical plate, as evidenced by *p*-values less than 0.05 showed that the model was significant statistically. To delve deeper into the influence of various parameters on the responses, contour and 3D surface, perturbation graphs were generated and scrutinised for each response (Fig. 4). In contrast to pure error, the residual plot shows that the *F* values of the various responses, which are 5.39 (R1), 78.36 (R2), 19.04 (R3), and 10.06 (R4), are negligible. Obtained fit statistics showed that *R*^2^ values of 0.9984 (R1), 0.9824 (R2), 0.8972 (R3) and 0.8778 (R4). Adjusted *R*^2^ values 0.912 (R1), 0.9699 (R2), 0.8638 (R3) and 0.7905 (R4). Predicted *R*^2^ values of 0.9831 (R1), 0.9128 (R2), 0.8539 (R3) and 0.6170 (R4) indicated a logical consensus between them (Table 5).

**Table 5:** ANOVA and regression summary of models

Responses	*F* Value	*P* Value	*R* ^2^	Adjusted *R*^2^	Predicted *R*^2^	Adequate precision	SD	CV (%)
Peak area of IRB	5.39	0.025	0.9984	0.9912	0.9831	7.1958	100.94	6.95
Retention time of IRB	78.36	<0.0001	0.9824	0.9699	0.9128	28.7612	0.12	4.52
Tailing factor of IRB	19.04	0.0004	0.8972	0.8638	0.8539	12.84	0.03	3.01
Theoretical plate numbers of IRB	10.06	0.0043	0.8778	0.7905	0.6170	11.7988	231.84	3.87

##### 3.2.3.1 Effects of independent factors on dependent factors

###### 3.2.3.1.1 Effect of independent factors on peak area

Model exhibited an *F* value of 6.49, indicating statistical significance with a *p*-value below 0.05. Furthermore, lack of fit resulted a *F* value of 0.34, also indicating statistical significance (Table 5). Fig. 4(a)–(c) depicts the impact of mobile phase ratio and flow rate on peak area through perturbation, 3-dimensional and contour plots. The peak area was negatively impacted by the mobile phase ratio and flow rate. Interestingly, we observed a notable increase in the flow rate as both flow rate and mobile phase increased. However, at lower levels, there was decreased in the peak area. Therefore, we determined that adjusting the mobile phase ratio and solvent flow rate could potentially produce a clear IRB peak which further aids in quantification. Eqn (4) illustrates the obtained linear polynomial equation for peak area.

4 Peak area of irbesartan = +1446.27 + 6.09547 × mobile phase – 600.65233 × flow rate

###### 3.2.3.1.2 Effect of independent factors on retention time

Model showed 78.36 *F* value, accompanied by a *p*-value below 0.05, underscores its statistical significance (Table 5). Similarly, the substantial *F*-value of 6.36 for lack of fit indicates significant influence wielded by the model. Fig. 4(d)–(f) portrays the impact of the flow rate and mobile phase on retention time through perturbation, 3D graphs, and contour plots for analysis. Consequently, the analytical method for IRB was devised by employing the maximum ratio to mitigate retention time. Notably, changes in mobile phase ratio yielded consistent retention times, indicating minimal influence. The flow rate emerges as a crucial factor directly influencing retention time. Elevated flow rates decreases retention time, whereas lower flow rates yield the opposite effect. The polynomial equation shows that rate of flow and ratio of mobile phase have a negative effect on the length of the IRB retention. However, their combined effect yields a positive outcome. Eqn (5) illustrates the obtained quadratic polynomial equation for retention time.

5 Retention time of irbesartan = + 22.9932 – 0.5922 × mobile phase – 5.5906 × flow rate – 0.0250 × mobile phase × flow rate + 0.0052 × mobile phase^2^ + 2.2140 × flow rate^2^

###### 3.2.3.1.3 Effect of independent factors on tailing factor

Model demonstrated a notable statistical effect yielding *F*-value of 19.04 (*p*-value < 0.05), as evident from Table 5. Additionally, the lack of fit *F* value of 0.72 showed a statistically significant lack of fit. A study was conducted to assess the impact of the flow rate and mobile phase ratio on the tailing factor. This investigation employed a polynomial equation alongside perturbation, 3-dimensional and contour plots (Fig. 4(g)–(i)). The findings revealed that the % of mobile phase exerted a significant influence on the tailing factor, with higher ratios correlating with a notable decrease in tailing. The tailing factor was negatively impacted by the flow rate. The polynomial equation showed how each component affected the tailing factor separately, with no apparent group effects. Furthermore, the model determined the statistical significance of the mobile phase ratio and flow rate. Eqn (6) illustrates the obtained linear polynomial equation for tailing factor.

6 Tailing factor of irbesartan = +1.50881 – 0.000539 × mobile phase – 0.404366 × flow rate

###### 3.2.3.1.4 Effect of independent factors on theoretical plates

Model yielded an *F* value of 10.06, indicating statistical significance (*p*-value < 0.05), underscoring its considerable impact. Additionally, the *F*-value of 1.67 signifies a statistically significant lack of fit, as depicted in Table 5. Utilising perturbation plots, 2D contour plots, 3D plots, and polynomial equations, a discernible quadratic correlation between variables and theoretical plates was elucidated (Fig. 4(j)–(l)). The data revealed a consistent rise in theoretical plates with decrease in mobile phase ratios, suggesting a preference for a lesser proportion of the mobile phase during method development. As the rate of flow increased from 0.4 to 0.8 mL min^−1^, the theoretical plates experienced a steady decline, resulting in a diminished plate count. This decline is deemed undesirable, prompting a reduction in the method's flow rate. Eqn (7) illustrates the obtained quadratic polynomial equation for theoretical plate number.

7 Theoretical plate number of irbesartan = +4953.4668 – 3.1048 × mobile phase – 4340.8170 × flow rate + 240.0000 × mobile phase × flow rate – 0.6319 × mobile phase^2^ – 6577.7876 × flow rate^2^

#### 3.2.4 Derringer's desirability

Optimal chromatographic parameters were ascertained through numerical optimisation, a technique involving the manipulation of various independent variables to attain specific objectives, like augmenting theoretical and plates peak area while diminishing tailing factor a and retention time. The aim is to achieve a desirability function value of one.^43^ In order to maximise the possibilities, the detected independent variables were kept within a range. Reducing the retention period of IRB yielded the best possible outcome while using the least amount of solvent. The most effective solution was achieved by combining a higher mobile phase ratio comprising 60% ethanol and 40% acetate buffer at a slower flow rate of 0.6 mL min^−1^. Under these optimised chromatographic conditions, the following results were attained: a peak area of 1451.61 milli absorbance units (mAU), a retention time of 2.73 minute, a theoretical plate count of 6159.57 centimeters and a tailing factor of 1.23. The cumulative desirability score was calculated to be 1000. The observed values for peak area, retention time, theoretical plate numbers and tailing factor were recorded as 1357.31 mAU, 2.83 minute, 6228.31 centimetres and 1.14 respectively. Upon comparing anticipated and attained values, all parameters exhibited *p*-values below 0.05. This indicates minimal variability and underscores a robust correlation between anticipated and actual outcomes within the experimental framework. The commendable chromatographic outcomes attained can be attributed to the systematic adoption of the AQbD methodology, which facilitates the development of optimal analytical solutions amidst competing objectives. This approach not only streamlined the enhancement of analytical results, but also elucidated the intricate relationship between causative factors and responses.

#### 3.2.5 Design space

**Fig. 5:**
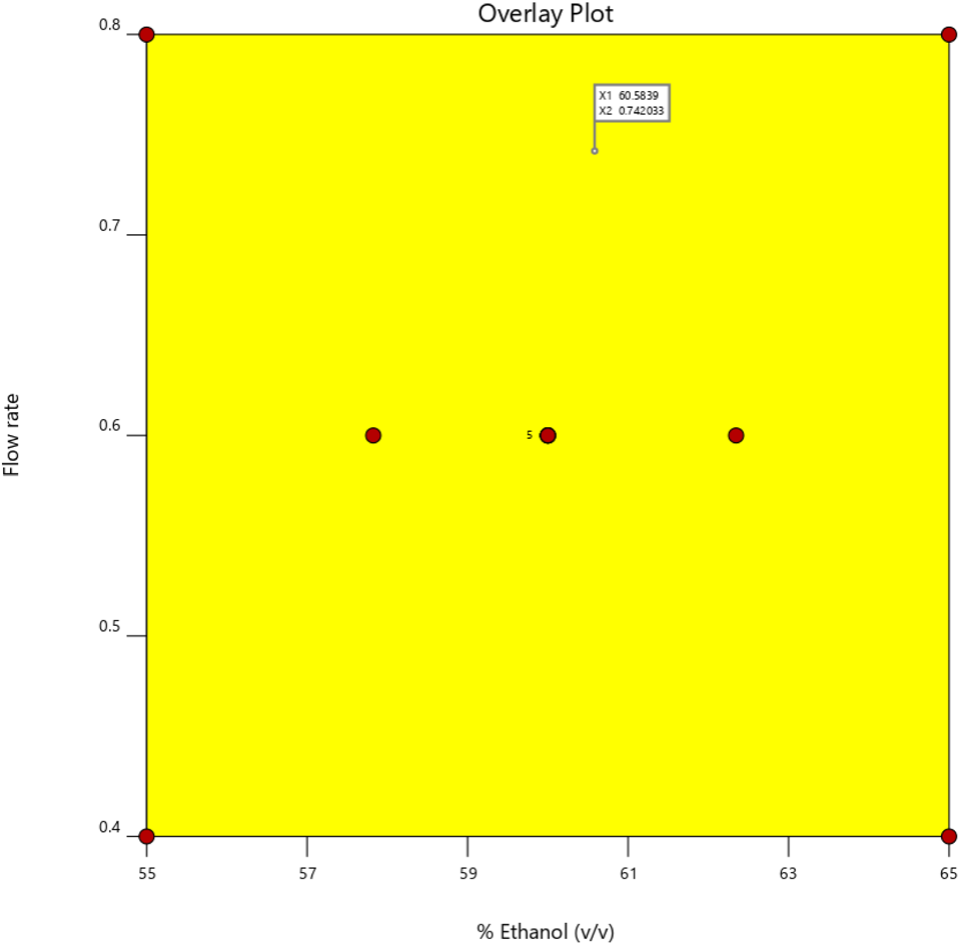
An overlay plot demonstrating the interaction of method's flow rate and % of ethanol.

To ensure the effectiveness of the HPLC technique, impact of CMPs on CAAs are carefully examined in the AQbD design space. The design space is represented by the “Yellow zone” in Fig. 5, which suggests that alterations within this region are unlikely to have an impact on the suggested approach's quality.

### 3.3 Validation of analytical procedure

#### 3.3.1 System suitability

Following 6 injections of 50 μg mL^−1^, the system suitability evaluation detected no noteworthy alterations in the fundamental parameters of IRB, comprising peak tailing factor, peak area, theoretical plates, and retention time. A concise overview of system suitability outcomes is presented in Table 6. The %RSD for all parameters remained below 2%, signifying that the recorded outcomes fell within the designated range.

**Table 6:** System suitability test parameters and results

Parameters	Mean ± SD (*n* = 6)	%RSD
Peak area of IRB	1428.16 ± 12.08	0.84
Retention time of IRB	2.83 ± 0.004	0.15
Tailing factor for IRB	1.23 ± 0.008	0.67
Theoretical plates of IRB	6207.16 ± 13.76	0.22

#### 3.3.2 Linearity

The calibration curve obtained illustrated the exceptional linearity of IRB across a spectrum of working standard solutions spanning from 80 to 120 μg mL^−1^. The coefficient of correlation (*R*^2^) was 0.9996, and the linear regression equation was *y* = 31.21*x* – 716.8.

#### 3.3.3 Limit of detection and limit of quantification

Sensitivity of the method was evaluated through the determination of LOD and LOQ, relying on the standard deviation (SD) of the response and the slope. The LOD and LOQ for IRB were found to be at 0.14 and 0.42 μg mL^−1^, underscoring its increased sensitivity level.

#### 3.3.4 Accuracy

The validity of the methodology was assessed through the recovery method. Control samples of low, medium, and high concentrations exhibited favourable recovery rates. The percentage relative standard deviation (%RSD) values were consistently below 2%, indicative of outstanding precision. Consequently, the findings remained within the acceptable range, underscoring the exceptional precision of the proposed IRB measurement technique. Refer to Table 7 for a comprehensive overview of the accuracy of the of the investigation results.

**Table 7:** Accuracy and precision data of intra-day and inter-day samples for the suggested HPLC technique, as evaluated at three IRB quality control levels

Levels	Concentration of standard taken (μg mL^−1^)	Concentration of sample added (μg mL^−1^)	%Recovery mean area ± SD (*n* = 6)	Intraday %RSD (*n* = 6)	Interday %RSD (*n* = 6)
LQC	90	100	100.46 ± 0.37	0.52	1.01
MQC	100	100	100.3 ± 0.1	1.12	1.32
HQC	110	100	100.1 ± 0.8	1.27	0.75

#### 3.3.5 Precision

The method's precision was evaluated through the analysis of three quality control standards: LQC, MQC, and HQC, with summarised outcomes provided in Table 7. Both inter-day and intra-day precision studies yielded %RSD values below 2%. This suggests that the obtained results fell within acceptable thresholds, highlighting the exceptional precision of the developed methodology.

### 3.4 Results of forced degradation investigations

**Fig. 6:**
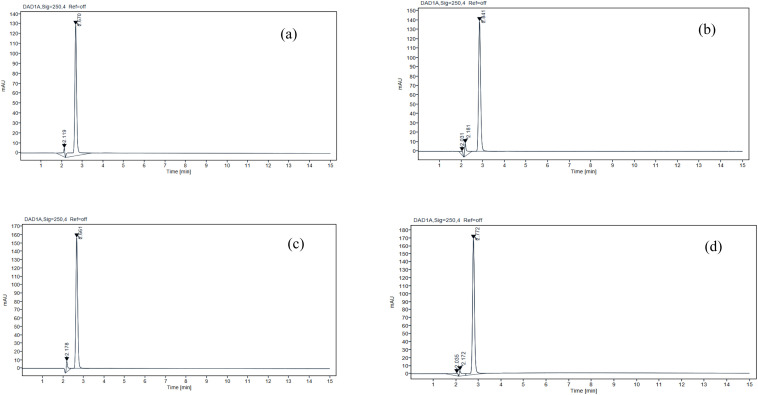
The chromatograms displaying IRB degradation peaks under the following conditions: (a) acid (b) alkali (c) peroxide and (d) thermal degradation.

The subsequent section presents a results of forced degradation experiments conducted on IRB. The primary deteriorating conditions encompassed exposure to 0.01 M hydrochloric acid solution, 0.01 M sodium hydroxide solution, 0.3% hydrogen peroxide solution and elevated temperature (60 °C). Upon exposure to acidic and alkaline environments, the compound IRB exhibited accelerated deterioration, as evidenced by the emergence of novel peaks at retention time 2.67 and 2.84 minute (Fig. 6a and b respectively). Under the influence of 0.3% hydrogen peroxide, IRB exhibited gradual degradation and exposure to heat-induced degradation has the mildest effect observed in the reduction of peak area (Fig. 6c and d respectively). The results of the degradation investigation are shown in Fig. 6, and the corresponding chromatograms are described in Table 8.

**Table 8:** Studies on the forced degradation of IRB

Type of degradation	Degradation condition	%Drug recovery	%Drug degradation
Acid degradation	0.01 M HCL/6 hours	88.8	11.2
Alkali degradation	0.01 M NaOH/6 hours	86.8	13.2
Peroxide degradation	0.3% H_2_O_2_/6 hours	93.57	6.43
Thermal degradation	80 °C/6 hours	98.45	1.55

### 3.5 Application of developed method in the characterization of irbesartan loaded polymeric nanoparticles

#### 3.5.1 Determination of %EE and %DL

The entropion efficiency (%EE) and drug loading (%DL) percentages of chitosan nano-particles were determined through a validated RP-HPLC method, quantifying the amount of IRB within the nanoparticles. Table 9 presents the findings regarding the presence of drug within nanoparticles. Chromatograms obtained show no excipients interference associated with chitosan nanoparticles in the solution containing the unentrapped drug. It was demonstrated that the proposed method for determination of drug loading and entrapment efficiency in the IRB-loaded polymeric nanoparticles was accurate and efficient.

**Table 9:** Characterization parameters of IRB loaded chitosan nanoparticles

Parameter	Irbesartan loaded polymeric nanoparticles
	Mean ± SD (*n* = 3)
Particle size (nm)	387.2 ± 0.5
PDI	0.15 ± 0.026
Entrapment efficiency (%)	73.52 ± 1.85
Loading efficiency (%)	12.38 ± 2.12

#### 3.5.2 *In vitro* release profile

**Fig. 7:**
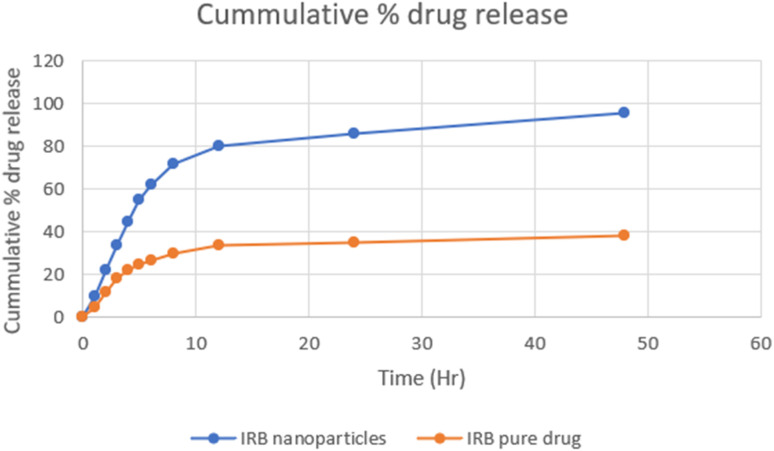
*In vitro* release profile of irbesartan and irbesartan-loaded chitosan nanoparticles.

The examination of the drug release kinetics from polymeric nanoparticles encapsulating IRB was carried out *in vitro* utilizing the dialysis bag technique (Fig. 7). Polymeric nanoparticles loaded with IRB exhibited a controlled release pattern, contrasting with the rapid release observed with pure IRB. The complete release of pure IRB occurred within three hours, whereas the release rate of IRB from chitosan nanoparticles reached 96.2% within a 42 hour period. Evaluating the drug release kinetics is crucial since the therapeutic impact of a drug is greatly influenced by how it releases from nanoparticles. Drug release can be further controlled by using kinetic models, which frequently assist in clarifying the release mechanism. In order to understand the mechanism of release, a range of mathematical models were utilized, such as first-order, zero-order, Korsmeyer-Peppas, Higuchi models (Table 10). The regression coefficient value *R*^2^ value 0.993 indicated that IRB-loaded polymeric nanoparticles release behavior conformed to zero-order. However, the Korsmeyer-Peppas model's value of “*n*,” was 0.312 and showed a Fickian diffusion release pattern that may have resulted from the breakdown of the nanoparticles, which created tiny pores that allowed the IRB to diffuse. This indicates that the simplified model assumes a constant diffusion flux independent of the concentration gradient.

**Table 10:** Release kinetics of chitosan nanoparticles loaded with IRB

Zero order (*R*^2^)	First order (*R*^2^)	Higuchi model (*R*^2^)	Korsmeyer-peppas model (*R*^2^)
0.9931	0.9423	0.9372	0.9912

### 3.6 Waste recycling

**Fig. 8:**
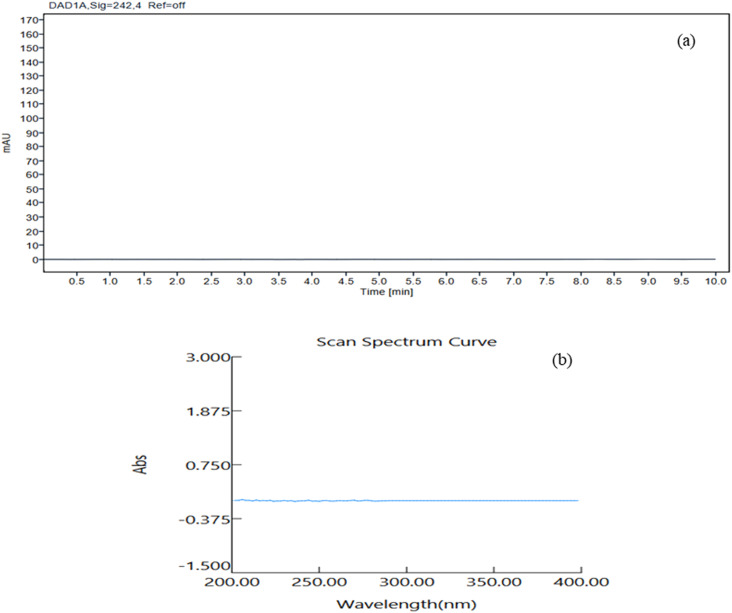
(a) HPLC chromatogram of distilled ethanol. (b) UV spectrum of the distilled ethanol using HPLC grade ethanol as a blank.

Method development and validation often use a significant amount of solvent, resulting in around 500 mL of waste, which includes the mobile phase used for in the column washing, analysis and other analytical objectives. The majority of this waste consists of around 70% ethanol and 30% buffer. To reduce environmental effects, we planned to recover waste ethanol using a simple distillation process, renewing the solvent and lowering pollution. Although ethanol is biodegradable, recycling adheres with the core principles of GAC. UV spectrophotometry was used to verify the purity of the distilled ethanol in the UV region with ethanol (HPLC grade) serving as blank (Fig. 8). HPLC analysis proved the absence of contaminants in distilled ethanol, which was then used for drug analysis. This strategy not only promotes environmental sustainability, but it also ensures that the analytical procedure is reliable and efficient.

### 3.7 Environmental impact assessment of the developed method

Contemporary scholars in academia are actively engaged in the advancement and enhancement of eco-friendly analytical methodologies while advocating for the adoption of less hazardous solvents over harmful alternatives. The paradigm GAC champions sustainability through the prioritisation of three core principles: substitution, reduction, and recycling. This entails the substitution of toxic solvents with environmentally benign alternatives or the reduction of their usage whenever feasible. An established method cannot simply be labelled as environmentally friendly without first undergoing extensive scrutiny with appropriate assessment tools. To determine the method's environmental sustainability, an in-depth analysis using relevant criteria is required. In this study, the procedure was evaluated using three assessment tools: the analytical GREEnness metric (AGREE), green analytical procedure index (GAPI), and analytical eco scale (AES). The data received from each assessment confirms the eco-friendliness and sustainability of the method. This evaluation ensures transparency and dependability in certifying the environmental credentials of the analytical approach. Table 11 compares the eco-friendliness of the published and proposed methods using GAPI, AGREE and AES tools.

**Table 11:** Comparison of previously published and suggested HPLC methods for green assessment

S.No	Reported by	Optimized chromatographic conditions	GAPI	AES	AGREE	Ref.
	M. M. Eswarudu *et al.*	Acetonitrile : sodium acetate buffer pH adjusted to 3.5 using ortho phosphoric acid, (55 : 45 v/v)	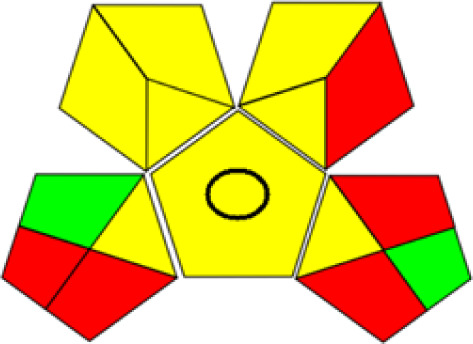	17 + 1 + 3 + 3 = 24	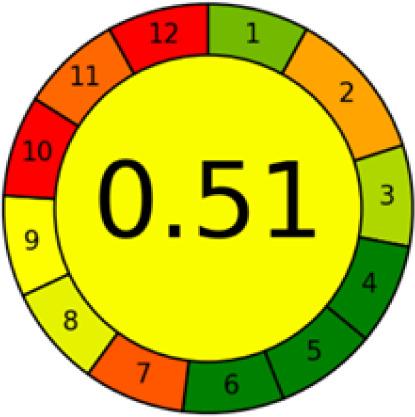	17
				AES = 76		
4	Kamala bodapati *et al.*	Potassium dihydrogen phosphate : acetonitrile, (70 : 30 v/v)	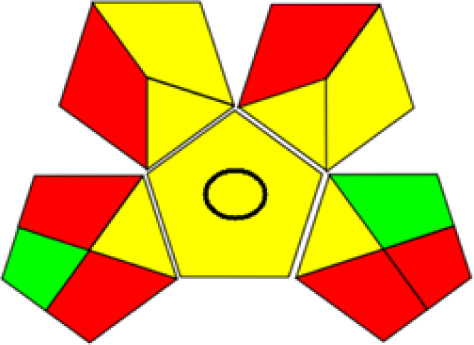	14 + 1 + 3 + 3 = 21	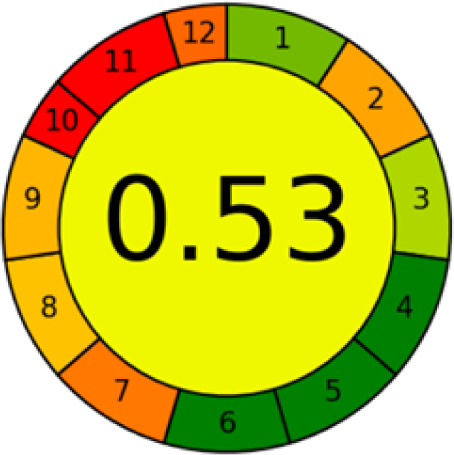	19
				AES = 79		
5	Tamer Awad Ali *et al.*	50 mM potassium di-hydrogen phosphate : acetonitrile, (55 : 45, v/v)	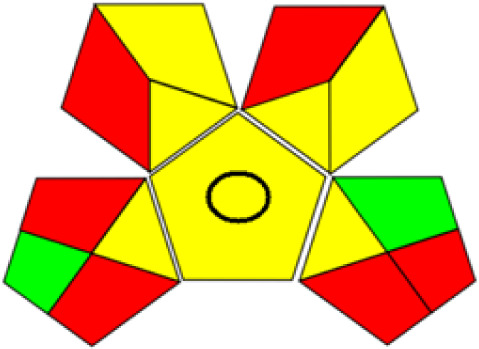	14 + 1 + 3 + 3 = 21	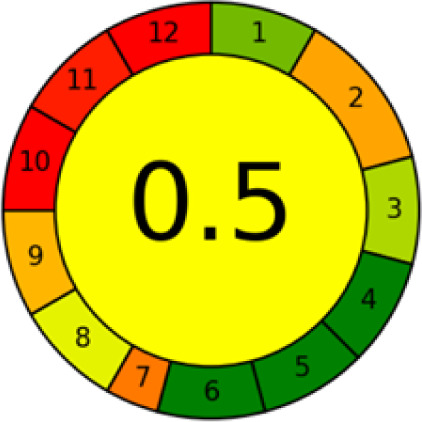	18
				AES = 79		
6	Reem Youssef *et al.*	Acetonitrile : phosphate buffer (pH 3), (40 : 60 v/v)	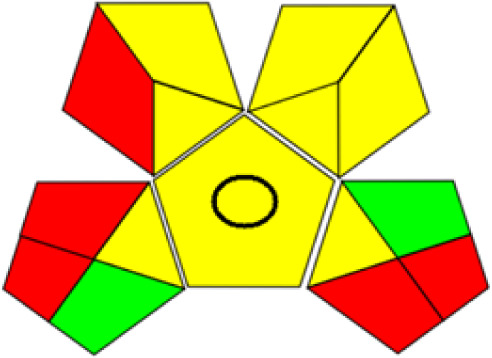	14 + 1 + 3 + 3 = 21	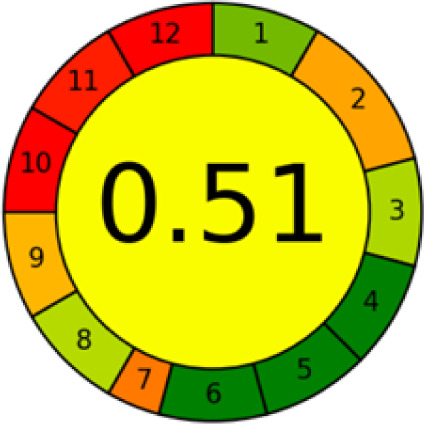	20
				AES = 79		
8	Rishabh K. Dagar *et al.*	Potassium dihydrogen phosphate buffer 0.05 M (pH 3.5) : acetonitrile : TEA, (80 : 20 : 0.1 v/v)	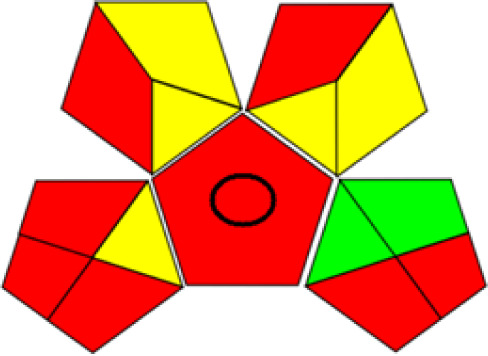	16 + 1 + 3 + 3 = 23	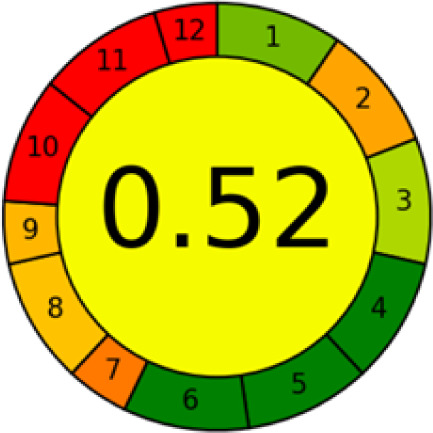	21
				AES = 77		
9	R. A. Mhaske *et al.*	0.05 M sodium dihydrogen phosphate buffer : acetonitrile (gradient mode)	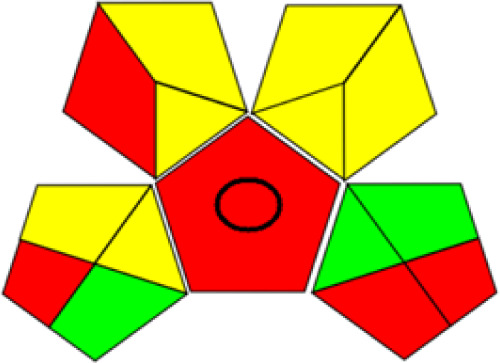	14 + 1 + 3 + 3 = 21	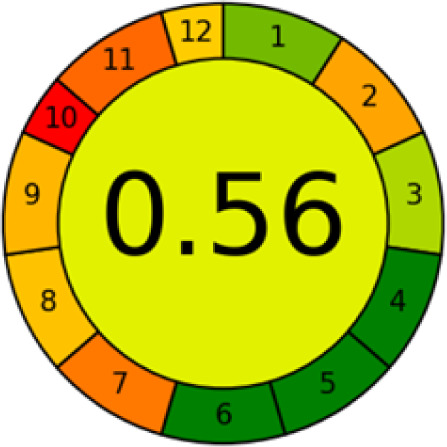	22
				AES = 79		
10	T. Hemant kumar *et al.*	Sodium acetate buffer pH 4.0 : acetonitrile, (30 : 70 v/v)	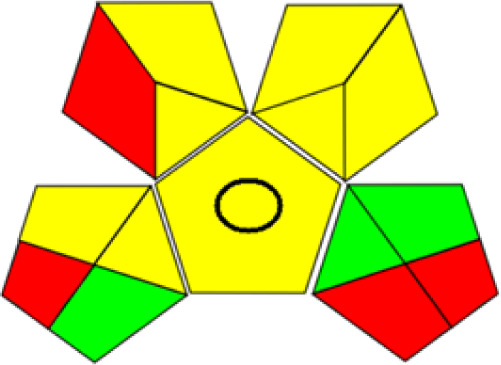	15 + 1 + 3 + 3 = 20	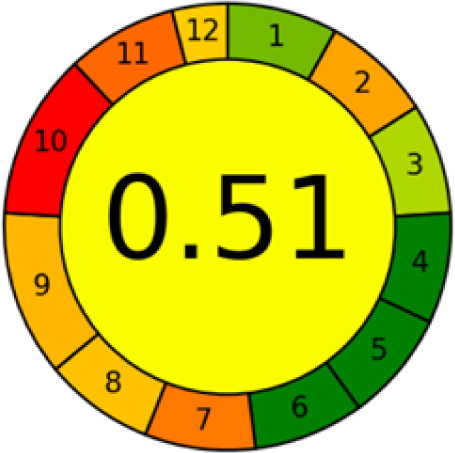	23
				AES = 80		
12	Hassan A. Alhazmi *et al.*	10 mM ammonium acetate buffer (pH 4.0) : acetonitrile, (40 : 60 v/v)	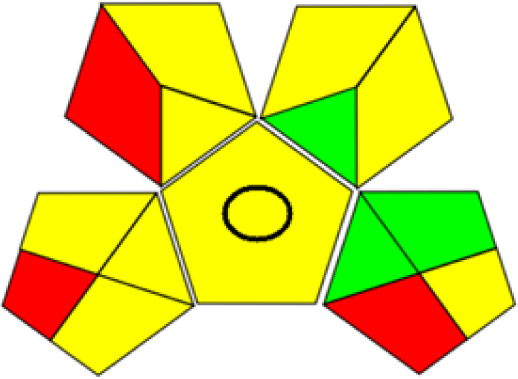	15 + 1 + 3 + 3 = 22	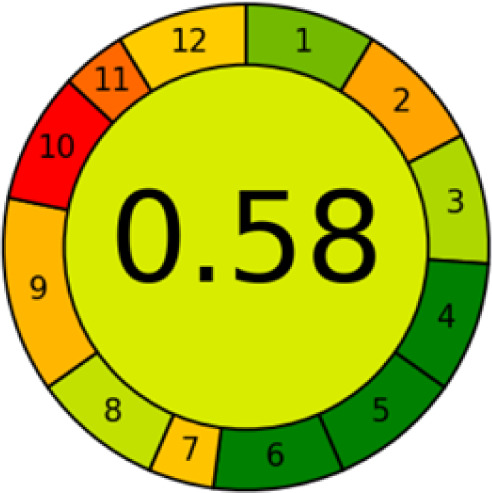	24
				AES = 78		
14	Proposed method	Ethanol : sodium acetate buffer, (60 : 40 v/v)	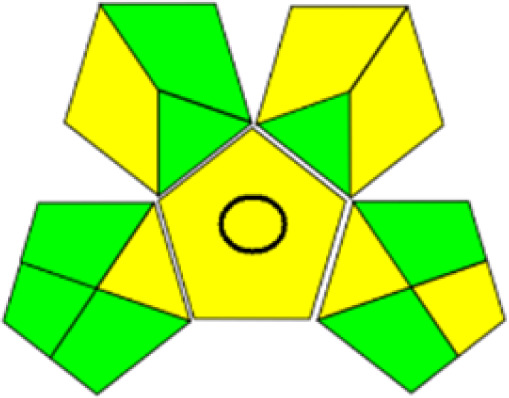	4 + 1 + 1 + 0	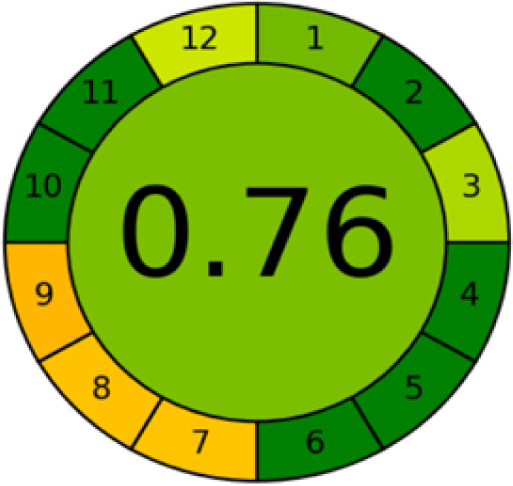	N/A
				AES = 94		

#### 3.7.1 GAPI

**Fig. 9:**
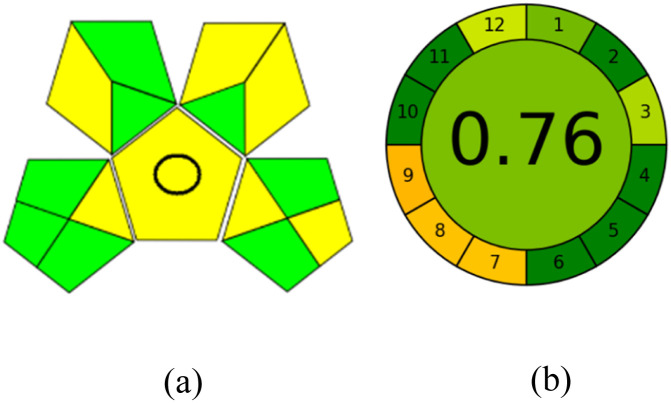
(a) GAPI, pictograms showing the developed method's green assessment findings. (b) AGREE metrics.

GAPI is divided into 11 categories, each coded with green, yellow and red to signal eco-friendliness, moderate risk and danger respectively. The eco-friendliness of the suggested method with ethanol was evaluated using GAPI pentagrams (Fig. 9a). The pentagram obtained consisted primarily of green and yellow sectors, indicating that the approach is environmentally favourable. The sample preparation pentagram indicated the absence of extraction and the use of green solvents during the preparation process. Furthermore, there was no need to preserve, store, or transport the samples. For the reagents, less than 10 mL was used for each analysis. Ethanol was classified as somewhat hazardous in terms of health dangers, but it posed no substantial safety problems.

The method was considered “green” in terms of instrumentation because LC uses less energy compared to other hyphenated techniques. The advantage of the developed method lies in recycling the used ethanol, thereby reducing waste. This extensive evaluation shows the method's compatibility with environmentally friendly and sustainable activities.

#### 3.7.2 AES

AES is a tool to assess procedures based on penalty points (PP). PP is calculated in four primary categories: reagents or chemicals used, instruments and their energy consumption, the quantity of the generated waste and occupational hazards for the analyst. The entire AES is then determined by applying the formula AES = 100 – PP. For this advanced method, the total penalty points were six, yielding an AES score of 94, signifying high sustainability and efficiency. The exact computation of AES is shown in Table 12.

**Table 12:** Calculation of AES for the proposed method

Calculation of solvents energy				
Solvents	Pictogram	GSH signal word	Amount of solvent	Subtotal
Ethanol	2	Danger	10–100 mL	4
Sodium acetate	1	Toxic	1	1
Water	0	0	0	

Calculation of instrumental energy PP				
Energy used	≤1.5 kW h per sample (HPLC)			1
Occupational hazard	The use of biosolvent and the hermitization process eliminates any occupational hazards			0
Waste management and recycling	0–10 mL			0
			Total PP	6
			AES	94

#### 3.7.3 AGREE metrics

The AGREE metric is the most recent geeness assessment instrument, covering all the 12 green analytical chemistry principles. Result is calculated using the scores of each individual principle, with values closer to one indicating more method greenness. After inputting the method details into the software, the results (Fig. 9b) showed that the approach is both environmentally benign and long-lasting.

## 4. Conclusion

Through a collaborative integration of Green Analytical Chemistry and AQbD methodologies, a successful RP-HPLC technique is developed for quantifying IRB. The method offers several advantages, including less retention time, expanded linearity range, enhanced precision and improved reproducibility. Central composite design was used to optimize of chromatographic conditions. The independent variables in the optimization process included the flow rate and mobile phase ratio, while the dependent variables encompassed number of theoretical plates, retention time, tailing factor, peak area. The outcomes of the CCD trials were meticulously recorded, and both graphical and numerical optimization techniques were employed. The optimized chromatographic conditions consisted of a 60 : 40% v/v ratio of ethanol and acetate buffer and a flow rate of 0.6 mL min^−1^. These conditions were further utilized for the detection and validation of IRB. A distinct drug peak was observed at 2.8 minute, with area of 1357 mAU, tailing factor and number of theoretical plates were found to be 1.14 and 6228.31 centimeters. The method's sensitivity was assessed through LOD and LOQ resulting in 0.14 μg mL^−1^ and 0.42 μg mL^−1^ respectively, indicating high sensitivity and the method also enables the detection of degradation products following exposure to stress conditions. Green assessment included the use of GAPI, AES, and AGREE metrics, which collectively confirmed superior environmentally friendly performance of the method. Overall, the proposed method surpasses previously reported methods and demonstrates minimal negative environmental impact. The comprehensive adoption and development of effective and ecologically sustainable AQbD methods for the analysis of pharmaceutical drugs utilising eco-friendly solvents could be the potential future consequences. Our proposed approach was employed to evaluate vitro drug release profile, drug loading and entrapment efficiency of IRB loaded polymeric nanoparticles. Notably, our method represents the first QbD strategy for quantifying IRB in polymeric nanoparticles. Furthermore, it facilitates the investigation of release kinetics across nanoparticles. This QbD-based method could also be used to quantify IRB in biological samples, which would make pharmacokinetic and bioequivalence studies easier.

## Data availability

The data that support the findings of this study are available on request from the corresponding author Kavitha Jayaseelan.

## Author contributions

Sinchana B Gopalaiah: conceptualization, data curation, formal analysis, investigation, methadology, validation, visualization, writing – original draft, writing – review and editing. Dr Kavitha Jayaseelan: project administration, supervision.

## Conflicts of interest

Authors declare no conflict of interest.
